# Supplementary material for: Dynamic MAIT cell response with progressively enhanced innateness during acute HIV-1 infection
Source: Nat Commun. 2020 Jan 14;11:272. doi: 10.1038/s41467-019-13975-9 (PMC6959336; doi:10.1038/s41467-019-13975-9)
Supplement: Supplementary file 1 — Supplementary Information [file 41467_2019_13975_MOESM1_ESM.pdf]

Dynamic MAIT cell response with progressively enhanced innateness during acute HIV-1 infection.

Lal et al.

Kerri Lal et al. Dynamic MAIT cell response with progressively enhanced innateness during acute HIV-1 infection. Supplementary material.

Supplementary Table 1. Related to Figure 1-7. RV217 Patient Demographics

Patient ID	Gender	Age (years)	Country	CD4 T cell nadir (cells/µl)	Time to peak VL (days)	Peak VL (log_{10} copies/ml)	Set point VL (log_{10} copies/ml)
10203	F	19	Uganda	843	12	5,57	3,89
10220	F	33	Uganda	796	18	5,49	3,69
10428	F	27	Uganda	421	10	6,92	4,08
10435	F	18	Uganda	392	14	6,74	3,52
10723	M	18	Uganda	414	6	6,32	4,35
10742	M	23	Uganda	286	14	7,94	5,20
20442	F	20	Kenya	364	11	6,54	3,57
30190	F	24	Tanzania	511	11	7,34	5,96
30812	F	19	Tanzania	306	14	7,53	4,85
30924	F	30	Tanzania	661	7	7,83	4,46
40007	M	25	Thailand	406	15	7,31	5,35
40032*	F	32	Thailand	615	0	6,55	4,95
40094	M	19	Thailand	866	16	6,40	4,64
40123	M	23	Thailand	616	14	6,24	4,47
40168	MtF	25	Thailand	444	11	6,66	5,28
40211*	M	18	Thailand	383	0	6,27	4,83
40231	MtF	21	Thailand	576	13	6,50	4,02
40250	M	35	Thailand	522	19	6,67	4,83
40257	MtF	18	Thailand	370	10	7,35	5,39
40265	M	23	Thailand	476	12	6,49	4,83
40353	M	21	Thailand	516	17	5,81	3,60
40363	MtF	29	Thailand	576	14	6,86	4,77
40436	MtF	29	Thailand	455	18	6,09	4,41
40491	MtF	18	Thailand	1052	10	6,91	4,30
40503*	M	23	Thailand	341	9	7,01	5,32
40511	MtF	27	Thailand	342	7	6,26	4,45
40512	MtF	18	Thailand	588	15	6,82	5,07
40577	M	23	Thailand	322	12	7,77	5,35
40646*	M	18	Thailand	324	14	7,46	4,81
Average		23		510	12	6,75	4,63

Peak = "True Peak", VL = HIV-1 viral load (copies/ml)
Set point VL = average of all measured viral loads between day 80 and day 365, in the absence of treatment (required at least two measurements)
CD4 nadir = minimum CD4 prior to day 80
* incident case without pre-infection time point

MtF = Male to Female Transgender

Supplementary Table 2. Related to Figure 1. RV254/SEARCH010 patient demographics

Patient ID	Gender	Age (years)	HIV status	CD4 count at visit	Viral Load at visit (Log10 copies/ml)
0486	M	31	Pos	378	4,61
0493	M	29	Pos	256	6,70
0539	M	23	Pos	262	6,84
0546	M	24	Pos	510	6,87
0557	M	41	Pos	552	6,90
0564	M	22	Pos	256	6,84
0565	M	25	Pos	346	5,56

Supplementary Table 3. **Related to Figures 1, 2, 5 and 7. RV217 MAIT cell phenotypic characteristics pre- and post- HIV-1 infection.**

	Pre-infection Interquartile range %+	Pre-infection Median %+	Post-infection Interquartile range %+	Post-infection Median %+	p value %+	Pre-infection Interquartile range MFI	Pre-infection Median MFI	Post-infection Interquartile range MFI	Post-infection Median MFI	p value MFI
**CD161**	96.1 - 99.4	98.7	96 - 99.5	98.8	0.97	613 - 1013	819	607 - 965	814	0.98
**CD8**	41.4 - 85.8	65.5	50.5 - 83.6	64.65	0.268	534 - 1122	688	492 - 1105	703.5	0.731
**CD4**	1.29 - 8.49	5.1	0.972 - 7.24	3.36	**0.007**	442 - 1225	659.5	400 - 1129	598	**0.031**
**CD8-CD4-**	9.86 - 50.9	28.2	13 - 43.3	29.25	**0.009**	N/A	N/A	N/A	N/A	N/A
**HLA-DR**	0.733 - 14.8	3.52	1.51 - 28.8	7.03	**<.001**	606 - 3561	1316	680 - 3263	1418	0.803
**PD-1**	0.0631 - 11.5	1.376	0 - 14.2	5.58	**<.001**	235 - 515	380	264 - 574	383	0.333
**CD38**	1.285 - 12	3.57	1.59 - 30.7	6.6	**<.001**	485 - 5703	606	471 - 6274	884	**0.033**
**TIGIT**	1.22 - 49.9	4.41	1.86 - 64.6	6.72	**0.005**	220 - 1278	543	226 - 1370	595	**0.004**
**Eomes**	36.6 - 82.6	65.7	38.8 - 75.9	68.8	0.463	314 - 596	360	320 - 667	374	**0.008**
**Grz B**	0.559 - 12.1	1.4	1.25 - 22	4.405	**<.001**	152 - 390	292.5	165 - 353	237.5	**0.035**
**CD56**	12.8 - 53.2	31.7	20.2 - 61.6	37.2	**0.002**	50.3 – 456	104.3	50.2 - 328	119	**0.019**
**CCR5**	68.8 - 96.5	93.6	87.8 - 99.3	95	**0.018**	85.1 - 227	158	141 - 238	203	**0.014**
**CCR6**	69.1 - 96.7	89.35	70.2 - 95.7	87.55	0.084	74.2 - 427	207.5	76.6 - 309	204.5	**0.049**
**Activated Caspase**	1.15 - 13.3	2.175	1.29 - 12.4	2.685	0.846	48.2 - 96.8	56.85	47.8 - 103	59	0.748
**IL12RB1**	6.72 - 71.7	42.15	13.8 - 79.4	55.65	**0.01**	42.5 - 90	68.75	52.1 - 103	78.55	**0.037**
**Ki-67**	0.371 - 2.36	0.8355	0.569 - 7.33	1.79	**0.039**	34.8 - 77.2	64.45	34.7 - 113	66.05	0.432

Lines 1-4, MAIT cells identified as 5-OP-RU loaded MR1 tetramer, **Lines 5-16** MAIT cell identified as CD161++Vα7.2+, **Lines 1-4, 9-11** n=17; **Lines 5-8** n=20; **Lines 12-16** n=10
Pre-infection: median days since first positive test for HIV-1 RNA = -109
Post-infection: median days since first positive test for HIV-1 RNA = 85, except Eomes and GrzB where median days since first positive test for HIV-1 RNA = 43
Grz B = Granzyme B

Supplementary Table 4. Related to Figure 3. Up and down regulated genes in post-infection timepoints compared to pre-infection from RNAseq data.

Up regulated genes compared to pre-infection

Peak VL		Set point VL			Early chronic infection	Shared upregulated genes at set point VL and peak VL	Shared upregulated genes at set point VL and early chronic infection	Shared upregulated genes at peak VL and early chronic infection	Shared upregulated genes at peak VL, set point VL, and early chronic infection
RAD51	CDCA8	CHI3L2			CHI3L2	CHI3L2	CHI3L2	CHI3L2	CHI3L2
CHI3L2	OASL	TPX2			IFNG	MYBL2	RRM2	DTL	RRM2
ACTR6	FGFBP2	MYBL2			IL1RL1	C3orf14		RRM2	
BIRC5	MRPL15	PDLIM1			PROX2	CDCA8			
CDC45	SLC38A6	ZNF330			DTL	FGFBP2			
CDC6	DTL	C3orf14			RFT1	CDCA7			
GZMH	CDCA7	MYB			RRM2	MAD2L1			
CDKN3	CCNA2	PKMYT1			IGHA1	PBK			
MYBL2	SPC25	TOP2A			ASS1P5	CDK1			
GDAP1	VBP1	CDCA8				RRM2			
NCAPG	CCNB2	FGFBP2							
RAD51AP1	PAQR4	CDCA7							
CD86	MAD2L1	CDCA5							
C3orf14	PTTG1	GPR180							
TXNDC9	SKA3	MAD2L1							
UCHL5	HPRT1	TMEM155							
CRYZ	IFI27	PBK							
CDC20	PCLAF	CDK1							
STMN1	CDT1	RRM2							
MND1	TK1	ZNF57							
ZWINT	PBK	FAM111B							
DLGAP5	CKAP2L	MIR425							
JCHAIN	BUB1	HNRNPRP2							
RAB33A	CDK1	MIR6077							
AURKB	RRM2								
APOBEC3B	CLEC12A								
PPA1	ADH6								
HIGD1A	UBE2C								
PLSCR1	TYMS								
CENPW	SDHD								
	DHFR								

Downregulated genes compared to pre-infection

Peak VL		Set point VL			Early chronic infection	Shared downregulated genes at set point VL and peak VL	Shared downregulated genes at set point VL and early chronic infection	Shared downregulated genes at peak VL and early chronic infection	Shared downregulated genes at peak VL, set point VL, and early chronic infection
ACTL6B	TMEM225	SELE	MITF	DUSP19	FNDC8	HRK	FNDC8	ACTBP8	RIMKLBP2
LZTS3	FAM221B	LRRC23	COLCA1	IGF2BP2-AS1	HIF3A	LINC00525	HIF3A	RIMKLBP2	SLC25A5P5
SLC23A2	C15orf59	XYLT2	KRT39	ZNF474	MTNR1B	CHRNA6	MTNR1B	SLC25A5P5	GAPDHP38
DEPDC5	EMP2	FNDC8	BCO2	CACNB2	RANBP6	TMEM225	COLCA1	GAPDHP38	
XYLT1	KRT18P33	MOCOS	ZNF813	PA2G4P2	CYSLTR2	C15orf59	OR10T1P		
SLC5A5	ACTBP8	CBX7	FAN1	GSN-AS1	SLC45A3	EMP2	CALML3-AS1		
CLCN2	NPTXR	PLEK2	RNA5SP302	ZBTB22	PINK1	RIMKLBP2	RIMKLBP2		
HRK	NPM1P19	TTC26	OR10T1P	EIF4A1P6	DCD	SLC25A5P5	SLC25A5P5		
USP20	RIMKLBP2	SLC1A2	ATP1A1-AS1	LINC02087	JUP	FBXW11P1	RPL31P45		
LINC00525	SLC25A5P5	SLCO1B3	TMEM225	RPL23AP32	SELP	RPL12P43	GAPDHP38		
CHRNA6	STK33P1	EFHD1	C15orf59	LINC01352	C14orf177	GSN-AS1			
TNNI1	TRIM60P18	IL1R2	CALML3-AS1	MTCYBP14	VSIG10	GAPDHP47			
NR2F6	LINC01494	TECTB	EMP2	PLCH1-AS1	ZNF385C	GAPDHP38			
NMNAT3	SFTA3	LRP11	RPS3AP36	KRT18P43	COLCA1	DUXAP1			
LALBA	RLIMP1	FKBP9	KCNU1	LSAMP-AS1	OR10T1P	LINC02185			
CCDC88B	RPL26P6	GPR83	ASS1P10	LINC02024	CALML3-AS1	HOMER3-AS1			
MROH2B	MTND5P7	HIF3A	TTTY5	DBIL5P2	SNORA70G	APOOP1			
CES4A	FBXW11P1	BTN2A3P	HMGB1P9	GAPDHP47	ACTBP8	VN1R76P			
GPR151	CFAP97D1	KIR2DL1	ATG3P1	RN7SL679P	RIMKLBP2				
GPR148	SPATA31B1P	GNG11	KRT41P	RN7SL483P	SLC25A5P5				
MST1	RPL12P43	LBP	RIMKLBP2	PGAM1P13	LINC01757				
CCDC121	GSN-AS1	MTNR1B	HAR1A	OR5M14P	SLC7A15P				
SMCR8	TARDBPP2	HRK	HMGB1P26	GAPDHP38	RPL31P45				
KLHL11	LINC02008	OR1J1	SLC25A5P5	SRD5A3-AS1	BTBD18				
OR52B1P	TCAM1P	ALDOB	TXNDC12-AS1	XBP1P1	ELF3-AS1				
OR52H1	ABCF2P1	C6orf52	RPSAP25	TMEM129	DNAJC9-AS1				
MGAT4EP	RPL6P9	TM4SF5	FBXW11P1	BNC1	OSTCP1				
BICD2	GAPDHP47	CELA2A	MIR4422HG	AHSP	KRT18P34				
MYO18A	MTND5P11	LINC00525	GAPDHP37	KRT20	GAPDHP38				
ENTPD7	MTND6P4	CHRNA6	HSD17B3-AS1	OR5AP2	FAM222A-AS1				
GRIN3A	GAPDHP38	SCHIP1	LPCAT2BP	SERPINE3	QRSL1P3				
LINC02185	HAUS8P1	MCHR2	RPL31P45	LINC01495	KRT18P18				
MYZAP	LINC02386	SETBP1	RPSAP13	PPIAP6					
RN7SL208P	DUXAP1	ADAMTSL3	RPL12P43	DUXAP1					
HOMER3-AS1	LINC01859	LRRC3	LINC01055	LINC01898					
APOOP1	VN1R76P	SCIMP	KRT24	LINC02185					
		SNORC	YWHAQP7	LINC01029					
		METTL7A	VN1R76P	HOMER3-AS1					
		ROR1	C18orf15	APOOP1					
		RPS4XP21	RN7SKP23	DDX43P2					
		HTR3D	OR4D9						

Supplementary Table 5. Related to Figure 3. Genes contributing to enrichment plots highlighted from GSEA data.

Negative regulation of viral entry into host cell enrichment plot

NAME	PROBE/GENE	RANK IN GENE LIST	RANK METRIC SCORE	RUNNING ES	CORE ENRICHMENT
row_0	IFITM2	56	0.765307069	0.15429236	Yes
row_1	SNX3	288	0.630943716	0.27820197	Yes
row_2	IFITM1	302	0.629056513	0.4056137	Yes
row_3	FCN1	707	0.548098028	0.5096298	Yes
row_4	TRIM5	736	0.545486569	0.6198168	Yes
row_5	IFITM3	1269	0.484781891	0.70870405	Yes

Interferon gamma production enchriment plot

NAME	PROBE/GENE	RANK IN GENE LIST	RANK METRIC SCORE	RUNNING ES	CORE ENRICHMENT
row_0	ISG15	176	0.669505477	0.033191286	Yes
row_1	IL1RL1	445	0.592546403	0.06056446	Yes
row_2	IFNAR1	573	0.5699476	0.08922745	Yes
row_3	LGALS9	1640	0.459312946	0.095129855	Yes
row_4	HLA-DPA1	1778	0.450283855	0.1171206	Yes
row_5	TLR3	1935	0.440222085	0.13822627	Yes
row_6	HLA-DRB1	2114	0.429899693	0.15837917	Yes
row_7	HAVCR2	2266	0.423427314	0.17866266	Yes
row_8	C1QBP	2330	0.419960469	0.20032841	Yes
row_9	HLA-DPB1	2654	0.404533535	0.21651718	Yes
row_10	KLRK1	2705	0.402339906	0.2374587	Yes
row_11	HMGB1	2800	0.397963643	0.25737754	Yes
row_12	IL27	2994	0.390398175	0.27511913	Yes
row_13	SLC11A1	3389	0.377358943	0.2885662	Yes
row_14	CD2	3526	0.37256974	0.3063575	Yes
row_15	HSPD1	3636	0.368771732	0.3244245	Yes
row_16	IL1B	3674	0.36729455	0.3436962	Yes
row_17	HLA-DRB5	4054	0.354829609	0.35618836	Yes
row_18	KLRC4-KLRK1	4318	0.347737223	0.37036568	Yes
row_19	CD244	4501	0.342252046	0.38569084	Yes
row_20	PYCARD	4745	0.335981905	0.39958715	Yes
row_21	HRAS	4957	0.330224901	0.4137421	Yes
row_22	GAS6	5004	0.329022348	0.43077627	Yes
row_23	SLAMF6	5526	0.315010071	0.4385735	Yes
row_24	CD14	6476	0.29130441	0.43744648	Yes
row_25	TNFSF9	6493	0.291006953	0.45295304	Yes
row_26	CEBPG	6662	0.287100136	0.4655351	Yes
row_27	SASH3	6676	0.286838442	0.48086897	Yes
row_28	RIPK3	7109	0.27643171	0.4881609	Yes
row_29	IL10	7132	0.275845051	0.5027376	Yes
row_30	FADD	7200	0.274314195	0.5164282	Yes
row_31	IL36RN	8754	0.242953509	0.5018987	Yes
row_32	TLR4	8920	0.239520669	0.5119523	Yes
row_33	LTA	9704	0.225956678	0.5102414	Yes
row_34	TICAM2	9859	0.222895622	0.51958907	Yes
row_35	ISL1	10109	0.21806626	0.5269794	Yes

Natural killer cell mediated immunity enrichment plot

NAME	PROBE/GENE	RANK IN GENE LIST	RANK METRIC SCORE	RUNNING ES	CORE ENRICHMENT
row_0	LYST	637	0.558890522	0.0745326	Yes
row_1	RAB27A	1143	0.495789021	0.14172097	Yes
row_2	PRDX1	1634	0.459863275	0.20365585	Yes
row_3	KLRD1	1932	0.440297127	0.266024	Yes
row_4	VAMP7	3519	0.372803718	0.29504502	Yes
row_5	TUBB	3593	0.370635092	0.35070056	Yes
row_6	GZMB	3809	0.362086326	0.40251136	Yes
row_7	MICB	3955	0.358021945	0.45494527	Yes
row_8	CORO1A	4127	0.353074402	0.50615543	Yes
row_9	RAET1E	5518	0.315214425	0.52982	Yes
row_10	ULBP1	6588	0.28888458	0.55516	Yes
row_11	CEBPG	6662	0.287100136	0.5979784	Yes
row_12	SLAMF7	7453	0.26904875	0.625243	Yes
row_13	PTPN6	7756	0.262802362	0.66024584	Yes
row_14	ULBP3	7912	0.259494781	0.6973605	Yes
row_15	KIR3DL1	7961	0.258278668	0.7361955	Yes

Supplementary Table 6. Related to Figure 4. Dominant MAIT cell TCR α and β chain clones in pre-infection and their expansion or contraction in chronic HIV infection.

	TRAV	TRAJ	TRBV	TRBD	TRBJ	Clone CDR3 sequence	Frequency pre-infection	Frequency post-infection	Classification
**Donor 1 10220**	TRAV1-2	TRAJ33				CAVMDSNYQLIW	38.90%	6.10%	Contract
**Donor 1 10220**			TRBV20-1	TRBD2	TRBJ2-1	CSARGLAGNEQFF	18.80%	12.40%	Contract
**Donor 2 10428**	TRAV1-2	TRAJ33				CAVRDSNYQLIW	30.80%	9.09%	Contract
**Donor 2 10428**			TRBV20-1	N/A	TRBJ1-2	CSARDIRTEFPGYTF	47.50%	33.20%	Contract
**Donor 3 10723**	TRAV1-2	TRAJ33				CAVRDSNYQLIW	22.60%	20.30%	Contract
**Donor 3 10723**			TRBV6-4	TRBD2	TRBJ2-3	CASSDGTSSTDTQYF	5.75%	N/A	N/A, likely contracted
**Donor 4 40353**	TRAV1-2	TRAJ33				CAVMDSNYQLIW	15.50%	1.50%	Contract
**Donor 4 40353**			TRBV20-1	TRBD1	TRBJ1-1	CSARDRDTEAFF	4%	2.70%	Contract
**Donor 5 40363**	TRAV1-2	TRAJ33				CAAMDSNYQLIW	14.70%	5.50%	Contract
**Donor 5 40363**			TRBV3-1/3-2	TRBD1	TRBJ2-5	CASSQDLRDRSLETQYF	7.30%	6.70%	Contract
**Donor 6 40436**	TRAV1-2	TRAJ33				CAVRDSNYQLIW	68.60%	61.40%	Contract
**Donor 6 40436**			TRBV27	TRBD1	TRBJ2-7	CASRNVRDVLGEQYF	45.20%	47.80%	Expand

Supplementary Table 7. **Related to Figures 2 and 5. Taqman gene expression assays from ThermoFisher used in Fluidigm Biomark targeted gene expression analysis**

Gene Name/Reagent	Source	Unique Identifier
BCL2	Bcl-2, apoptosis regulator	Assay ID# Hs00608023_m1
BCL2A1	Bcl-2 related protein A1, apoptosis regulator	AssayID# Hs00187845_m1
BIRC3	Baculoviral IAP repeat containing 3	Assay ID# Hs00985031_g1
CASP1	Caspase 1	Assay ID# Hs00354836_m1
CASP3	Caspase 3	Assay ID# Hs00234387_m1
CCL3	MIP-1a, C-C motif chemokine ligand 3	Assay ID# Hs00234142_m1
CCL4	MIP-1b, C-C motif chemokine ligand 4	Assay ID# Hs99999148_m1
CCL5	RANTES, C-C motif chemokine ligand 5	Assay ID# Hs00174575_m1
CCNA2	Cyclin A2	Assay ID# Hs00996788_m1
CCND2	Cyclin D2	Assay ID# Hs00153380_m1
CCR1	CD191, C-C motif chemokine receptor 1	Assay ID# Hs00928897_s1
CCR6	CD196, C-C motif chemokine receptor 6	Assay ID# Hs01890706_s1
CCR7	CD197, C-C motif chemokine receptor 7	Assay ID# Hs01013469_m1
CD160	CD160 molecule	Assay ID# Hs00199894_m1
CD161, KLRB1	Killer cell lectin like receptor B1	Assay ID# Hs00174469_m1
CD16A	FcyRIIIa, low affinity Fc receptor	Assay ID# Hs00275547_m1
CD226	CD226 molecule, DNAM-1	Assay ID# Hs00170832_m1
CD244	CD244 molecule, 2B4	Assay ID# Hs00900271_m1
CD274	CD274 molecule, PDL1	Assay ID# Hs01125301_m1
CD3	CD3d molecule	Assay ID# Hs00174158_m1
CD38	CD38 molecule	Assay ID# Hs01120071_m1
CD4	CD4 molecule	Assay ID# Hs01058407_m1
CD69	CD69 molecule, CLEC2C	Assay ID# Hs00934033_m1
CD8	CD8a molecule, Leu2	Assay ID# Hs00233520_m1
CDK6	Cyclin dependent kinase 6	Assay ID# Hs01026371_m1
CRTAM, CD355	Cytotoxic and regulatory T-cell molecule	Assay ID# Hs00219699_m1
ENTPD1	Ectonucleoside triphosphate Diphosphohydrolase 1, CD39	Assay ID# Hs00969559_m1
EOMES	Eomesodermin	Assay ID# Hs00172872_m1
FAS	Fas cell surface death receptor, CD95	Assay ID# Hs00531110_m1
FASLG	Fas ligand, CD178	Assay ID# Hs00181225_m1
GNLY	Granulysin, LAG-2	Assay ID# Hs00246266_m1
GZMA	Granzyme A, CTLA3	Assay ID# Hs00989184_m1
GZMB	Granzyme B, CTLA1	Assay ID# Hs00188051_m1
GZMK	Granzyme K, TRYP2	Assay ID# Hs00157878_m1
HLADRA	Major histocompatibility complex, class II, DR alpha	Assay ID# Hs00219578_m1
ICOS	Inducible T-cell costimulator, CD278	Assay ID# Hs00359999_m1
IFI16	Interferon gamma	Assay ID# Hs00989291_m1
IFNG	Interferon gamma inducible protein 16	Assay ID# Hs00194261_m1
IGF2R	Insulin like growth factor 2 receptor, CD222	Assay ID# Hs00974474_m1
IKZF1	IKAROS family zinc finger 1	Assay ID# Hs00958474_m1
IKZF2	IKAROS family zinc finger 2, HELIOS	Assay ID# Hs00212361_m1
IL12RB1	Interleukin 12 receptor subunit beta 1, CD212	Assay ID# Hs00538167_m1
IL12RB2	Interleukin 12 receptor subunit beta 2	Assay ID# Hs01548202_m1
IL18R1	Interleukin 18 receptor 1, IL18RA	Assay ID# Hs00977691_m1
IL18RAP	Interleukin 18 receptor accessory protein, IL-18Rbeta	Assay ID# Hs00977695_m1
IL21R	Interleukin 21 receptor, CD360	Assay ID# Hs00222310_m1
IL2RA, CD25	Interleukin 2 receptor subunit alpha	Assay ID# Hs00907778_m1
IL2RG	Interleukin 2 receptor subunit gamma, CD132	Assay ID# Hs00953624_m1
IL7R	Interleukin 7 receptor, CD127	Assay ID# Hs00902334_m1
IRF4	Interferon regulatory factor 4	Assay ID# Hs01056533_m1
IRF7	Interferon regulatory factor 7	Assay ID# Hs01014809_g1
KIR3DL1, KIR3DS1	Killer cell immunoglobulin like receptor, three Ig domains and long cytoplasmic tail 1	Assay ID# Hs00744448_s1
KLF10	Kruppel like factor 10	Assay ID# Hs00921811_m1
KLRC1	Killer cell lectin like receptor C1, NKG2A	Assay ID# Hs00970273_g1
KLRC2, KLRC3	Killer cell lectin like receptor C2, NKG2C	Assay ID# Hs04192492_gH
KLRD1	Killer cell lectin like receptor D1, CD94	Assay ID# Hs00233844_m1
KLRF1	Killer cell lectin like receptor F1	Assay ID# Hs00212979_m1
KLRG1	Killer cell lectin like receptor G1	Assay ID# Hs00929964_m1
KLRK1,KLRC4,KLRK1	Killer cell lectin like receptor K1, NKG2D	Assay ID# Hs00183683_m1
LAIR1	Leukocyte associated immunoglobulin like receptor 1, CD305	Assay ID# Hs00253790_m1
LAMP1,CD107	Lysosomal associated membrane protein 1	Assay ID# Hs00174766_m1
MAP3K8	Mitogen-activated protein kinase kinase kinase 8	Assay ID# Hs00178297_m1
MIR155,MIR155HG	MIR155 host gene	Assay ID# Hs01374569_m1
MKI67	Ki-67, cell proliferation	Assay ID# Hs01032433_m1
MTHFD2	Methylenetetrahydrofolate dehydrogenase (NADP+ dependent) 2, methenyltetrahydrofolate cyclohydrolase	Assay ID# Hs00759197_s1
NCAM1, CD56	Neural cell adhesion molecule 1	Assay ID# Hs00941830_m1
NCR1	Natural cytotoxicity triggering receptor 1, CD335	Assay ID# Hs00183118_m1
NCR3	Natural cytotoxicity triggering receptor 3, CD337	Assay ID# Hs00394809_m1
NFKB1	Nuclear factor kappa B subunit 1, NF-kB1	Assay ID# Hs00765730_m1
NFKBIA	NFKB inhibitor alpha	Assay ID# Hs00355671_g1
NFKBID	NFKB inhibitor delta	Assay ID# Hs01076336_m1
NKG7	Natural killer cell granule protein 7	Assay ID# Hs01120688_g1
PDCD1	Programmed cell death 1, PD-1, CD279	Assay ID# Hs01550088_m1
PLZF, ZBTB16	Zinc finger and BTB domain containing 16	Assay ID# Hs00957433_m1
SERPINB9	Serpin family B member 9	Assay ID# Hs00394497_m1
SLAMF5	CD84 molecule	Assay ID# Hs01547121_m1
SLAMF7	SLAM family member 7, CD319	Assay ID# Hs00900280_m1
STX11	Syntaxin 11	Assay ID# Hs01891623_s1
TBX21	T-box 21, T-bet	Assay ID# Hs00203436_m1
TESPA1	Thymocyte expressed, positive selection associated 1	Assay ID# Hs00207702_m1
TGFBR1	Transforming growth factor beta receptor 1	Assay ID# Hs00610318_m1
TIGIT	T-cell immunoreceptor with Ig and ITIM domains	Assay ID# Hs00545087_m1
TNF	Tumor necrosis factor, TNF-alpha	Assay ID# Hs01113624_g1
TNFRSF9	TNF receptor superfamily member 9, CD137	Assay ID# Hs00155512_m1
TNFSF10	Tumor necrosis factor superfamily member 10, TRAIL	Assay ID# Hs00921974_m1

Supplementary Table 8, Related to Figures 1, 2, 5, 6 and 7.

Reagent	Source	Identifier	Dilution used
MR1 5-OP-RU PE human tetramer	NIH Tetramer Facility		1/500
mouse anti-human CD8 PerCP-Cy5.5	BD Biosciences	Cat#341051; Clone SK1; RRID:AB_400209	1/10
mouse anti-human CD161 Brilliant Violet 605	BioLegend	Cat#339916; Clone HP-3G10; RRID#AB_2563607	1/40
mouse anti-human HLA-DR Brilliant Violet 650	BioLegend	Cat#307650; Clone L243; RRID#AB_2563828	1/40
mouse anti-human CD38 Brilliant Violet 711	BioLegend	Cat#303528; Clone HIT2; RRID#AB_2563811	1/20
mouse anti-human PD-1 BD Horizon BV786	BD Biosciences	Cat#563789; Clone EH12.1; RRID:AB_2738425	1/80
mouse anti-human Vα7.2 TCR APC	BioLegend	Cat#351708; Clone 3C10; RRID#AB_10933246	1/40
mouse anti-human CD14 Alexa Fluor 700	BD Biosciences	Cat#557923; Clone M5E2; RRID:AB_396944	1/20
mouse anti-human CD19 Alexa Fluor 700	BD Biosciences	Cat#557921; Clone H1B9; RRID:AB_396942	1/160
mouse anti-human CD4 APC-H7	BD Biosciences	Cat#641398; Clone SK3; RRID:AB_1645732	1/40
mouse anti-human CD3 PE-Texas Red	Thermo Fisher	Cat#MHCD0317; Clone S4.1; RRID#AB_10376002	1/40
mouse anti-human TIGIT PE-Cy7	eBioscience	Cat#20-9500-42; Clone MBSA43; RRID#AB_2573548	1/20
mouse anti-human CD56 PE-Cy7	BD Biosciences	Cat#557747; Clone B159; RRID:AB_396853	1/80
mouse anti-human CCR5 BUV737	BD Biosciences	Cat#565293; Clone 2D7/CCR5; RRID:AB_2739162	1/160
mouse anti-human CCR6 BV650	BD Biosciences	Cat#563922; Clone 11A9; RRID:AB_2738488	1/20
mouse anti-human IL12RB1 BV786	BD Biosciences	Cat#744207; Clone 2.4E6; RRID:AB_2742059	1/20
mouse anti-human EOMES FITC	ThermoFisher	Cat# 11-4877-42; Clone WD1928; RRID#AB_2572499	1/20
mouse anti-human Ki67 BUV395	BD Biosciences	Cat#564071; Clone B56; RRID:AB_2738577	1/20
mouse anti-human/mouse Granzyme B Pacific Blue	BioLegend	Cat#515408; Clone GB11; RRID#AB_2562196	1/20
mouse anti-human CD107a BUV395	BD Biosciences	Cat#565113; Clone H4A3; RRID:AB_2739073	1/20
mouse anti-human TNF BV650	BD Biosciences	Cat#563418; Clone MAb11; RRID:AB_2738194	1/20
mouse anti-human IFNγ BV786	BD Biosciences	Cat#563731; Clone 4S.B3; RRID:AB_2738391	1/80
mouse anti-human CD28 purified	BioLegend	Cat#302902, Clone CD28.2; RRID#AB_314304	1/800

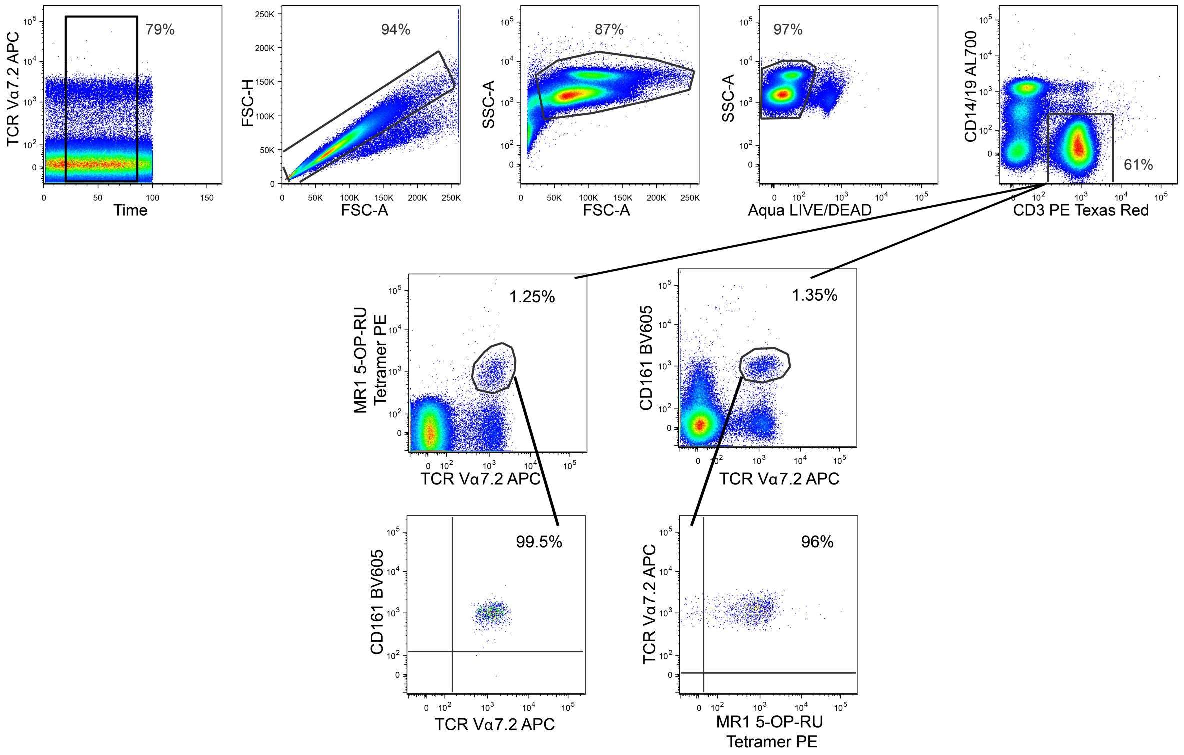

Supplementary Fig. 1. Related to Figures 1 - 7. Example polychromatic flow cytometry gating strategy for identification of MAIT cells in cryopreserved PBMC samples in the acute HIV-1 capture cohort. Cryopreserved PBMC were stained with monoclonal antibodies or 5-OP-RU loaded MR1 tetramer for the identification of MAIT cells within CD3+, living T cells. In cases where MAIT cells were sorted for Fluidigm Biomark gene expression analysis or RNA-seq, no time gating was included.

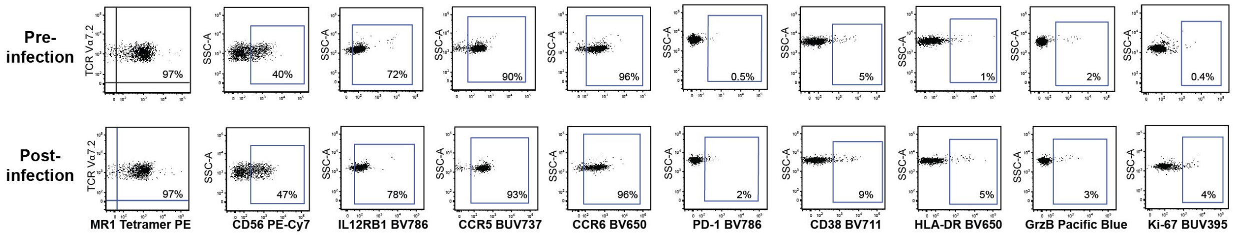

Supplementary Fig. 2. Related to Figures 1, 2, 5 and 7. Example staining of phenotypic and transcriptional markers in one pre-infection and one post-infection time point. MAIT cells identified as CD161++Vα7.2+ of CD3+ T cells were gated on in one pre-infection (days since first positive test for HIV-1 RNA = -142) and one post-infection (days since first positive test for HIV-1 RNA = 41) time point.

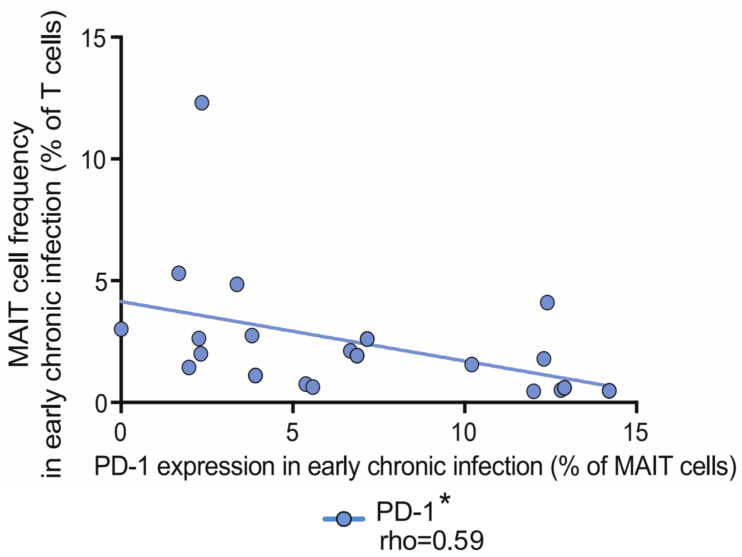

Supplementary Fig 3. Related to Figures 1 and 2. Surface expression of PD-1 as measured through flow cytometry on MAIT cells correlates with the frequency of blood MAIT cells. *p≤0.05

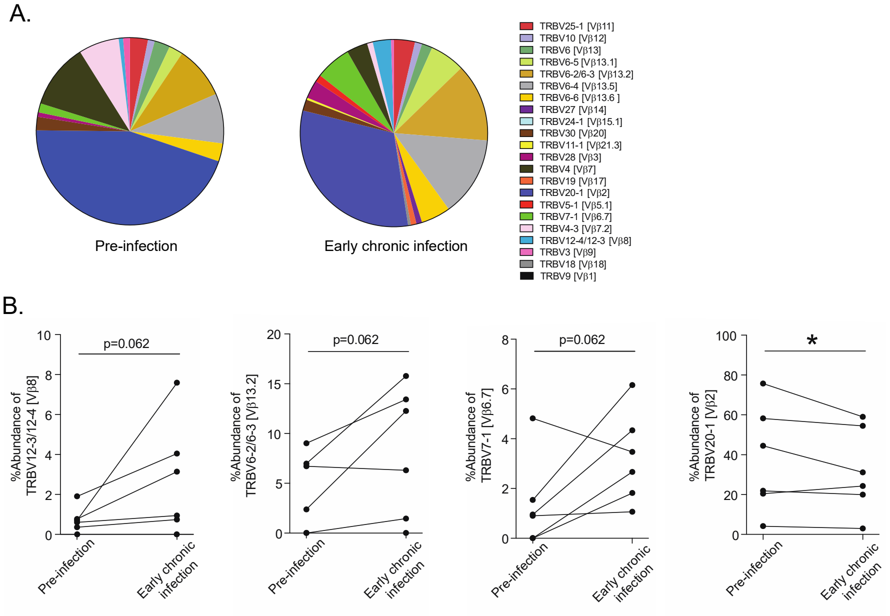

Supplementary Fig. 4. Related to Figure 4. TRBV usage in MAIT cells in pre-infection and early, chronic HIV infection. Bulk sorted MAIT cells identified as Vα7.2+ CD161++ of CD3+ T cells were subjected to RNA-seq for TCR analysis via the MiXCR platform. (**A**) Representative pie chart displaying TRBV usage in MAIT cells for one donor (40353) and (**B**) the percent abundance of four TRBV segments in pre-infection and the early, chronic post-infection time point.

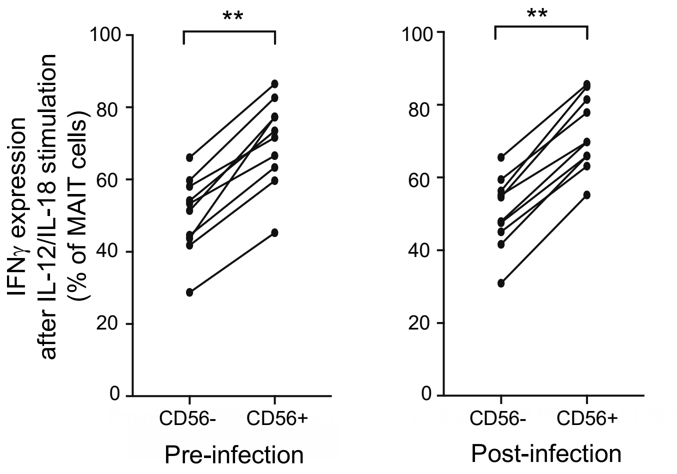

Supplementary Fig 5. Related to Figure 5. **CD56+ MAIT cell produce more IFNγ in response to IL-12/IL-18 stimulation than their CD56- counterparts in pre- and post-HIV infection.** Intracellular IFNγ production measured after IL-12/IL-18 stimulation in CD56- vs. CD56+ MAIT cells in donors with matched pre-infection and post-infection (approximately 85 days post-infection) time points.

Supplementary Methods

MAIT cell frequency, activation, phenotype, and cell sorting flow cytometry panel. Three polychromatic flow cytometry panels were used to measure MAIT cell frequency and phenotype in cryopreserved peripheral blood mononuclear cells (PBMC). Briefly, thawed samples were washed, stained with LIVE/DEAD Fixable Aqua Dead Cell dye (ThermoFisher) blocked for Fc receptors using Normal mouse serum (ThermoFisher) and surface stained with antibody cocktail. 20 donors with longitudinal samples from one pre-infection and three post-infection time points corresponding to peak VL, set point VL, and early chronic infection were surface stained as previously described (*1*). MAIT cells from all 20 donors were FACS sorted for targeted transcriptomics (Aria SORP, BD Biosciences), and 9 donors were FACS sorted for RNA-seq analysis. The phenotyping and surface panel included CD14 AlexaFluor700 (clone M5E2, BD Biosciences), CD19 AlexaFluor700 (clone H1B19, BD Biosciences), CD3 PE Texas Red (clone S4.1, ThermoFisher), CD4 APC-H7 (clone SK3, BD Biosciences), CD8 PerCP-Cy5.5 (clone SK1, BD Biosciences), CD161 BV605 (clone HP-3G10, BioLegend), Vα7.2 APC (clone 3C10, BioLegend), HLA-DR BV650 (clone L243, BioLegend), CD38 BV711 (clone HIT2, BioLegend), PD-1 BV785 (clone EH12.1, Biosciences), TIGIT PE-Cy7 (clone MBSA43, ThermoFisher). Cryopreserved PBMC from 29 donors (9 additional from the above staining) were surveyed for CD56 expression and MR1 tetramer confirmation of the CD161++Vα7.2+ MAIT cell population, which included one pre-infection time point and up to 10 post-infection time points in acute HIV infection, ranging from 1-1,009 days post first positive test for HIV-1 RNA. These samples were stained with several of the antibodies listed above, as well as surface staining with the PE-conjugated 5-OP-RU loaded MR1 tetramer (NIH Tetramer Core Facility) and CD56 PE-Cy7 (clone B159, BD Biosciences). Finally, 10 cryopreserved PBMC samples were stained for chemokine expression in MAIT cells in acute HIV infection, with one pre-infection and one post-infection time point (median days since first positive test for HIV-1 RNA=85) which included CCR5 BUV737 (clone 2D7/CCR5, BD Biosciences), CCR6 BV650 (clone 11A9, BD Biosciences), and IL12RB1 BV786 (CD212, clone 2.4E6, BD Biosciences).

MAIT cell functional assay. MAIT cell functionality pre- and post-HIV infection was assessed using three stimulation techniques. Bulk PBMC were stimulated for either 24-hours with partially fixed *E. coli* D21 (MOI: 0.1) in the presence of anti-CD28 (clone 37.51, BioLegend) and CD107a PE-Cy7 or BUV395 (clone H4A3, BD Biosciences) for 24-hours with IL-12 p70 (10 ng/mL, Peprotech) and IL-18 (100 ng/mL, Medical & Biological Laboratories) as previously described (*2*), or for 6 hours with PMA/ionomycin (eBioscience™ Cell Stimulation Cocktail (500X), ThermoFisher) per manufacturers recommendation. All stimulation methods included BFA (eBioscience™ Brefeldin A Solution (1000X), ThermoFisher) and monensin (BD GolgiStop containing monensin, BD Biosciences) for the last 6 hours of stimulation. After stimulation, PBMC from some donors were stained for activated caspases (Violet Live Cell Caspase Probe, BD Biosciences). All PBMC were stained for living cells with Aqua LIVE/DEAD viability dye (ThermoFisher), Fc receptors were blocked with normal mouse serum (ThermoFisher), and surface stained for 30 minutes at room temperature. After fixation with 2% paraformaldehyde, MAIT cells were stained for intracellular cytokines for 30 minutes with Perm/Wash Buffer (BD Biosciences). Anti-human antibodies used to stain for intracellular cytokines included IFNγ

BV785 (clone 4S.B3, BioLegend), TNF BV650 (clone MAb11, BD Biosciences), and Granzyme B (GrzB) Pacific Blue (clone GB11, BioLegend).

Fluidigm BioMark for targeted transcriptomics on sorted MAIT cells. Cryopreserved PBMC from 20 donors in 4 donor-matched time points corresponding to 1 pre-infection and 3 post-infection time points correlating with peak viral load, set point viral load, and early chronic infection were used for targeted transcriptomics using the Fluidigm Biomark platform. PBMC were thawed, washed with PBS, and stained with aminereactive viability dye (Live/Dead Aqua, Invitrogen) for 30 minutes at RT. Cells were washed with staining buffer (PBS and 0.5% BSA) and Fc receptors were blocked for 15 minutes at RT with Normal Mouse IgG (Invitrogen). Cells were then stained for 30 minutes at RT with CD3 PE Texas Red (Clone S4.1, ThermoFisher), CD14 AlexaFluor700 (Clone M5E2, BD Biosciences), CD19 AlexaFluor700 (Clone HIB19, BD Biosciences), CD8 PerCP-Cy5.5 (clone SK1; BD Biosciences), CD4 APC-H7 (Clone SK3, BD Biosciences), CD161 Brilliant Violet 605 (clone HP-3G10, BioLegend), and TCR Vα7.2 APC (Clone 3C10, BioLegend). After being washed twice with staining buffer, PBMCs were sorted using a FACSAria (BD Biosciences) running on FACSDiVa software (BD Biosciences). Cells for sorting were gated on through doublet exclusion, Aqua Live/Dead negative, CD3+, CD14/CD19 negative cells. MAIT cells were identified as CD161^{bright}, TCR Vα7.2+, and were sorted at 100 cells per well into a 96-well plates containing a reverse transcription pre-amplification reaction mix, which contains 0.2X assay mix (85 pre-selected primers and probes (ThermoFisher), Superscript III platinum Taq (ThermoFisher), and SUPERase-In RNase Inhibitor (ThermoFisher). The reverse transcription reaction was performed as 1 cycle of 15 minutes at 50°C followed by 2 minutes at 95°C, and pre-amplification was 16 cycles of 95°C for 15 seconds, and 60°C for 4 minutes. cDNA produced following this step was diluted 1:5 in DNA suspension buffer (Teknova). Diluted cDNA was combined with TaqMan Universal PCR Master mix (ThermoFisher) and further diluted with 20X GE sample loading reagent (Fluidigm), and loaded into one side of a primed, 96.96 Dynamic Array chip (Fluidigm). Experimental assays (20X assay mixes of TaqMan primers and probes) were diluted 1:1 with 2X assay loading buffer (Fluidigm), and loaded onto the other side of the chip. The chip was then loaded into an IFC Controller (Fluidigm) to fill the chip matrix with sample and assay. The chip was transferred to a Biomark (Fluidigm) for thermocycling and fluorescence acquisition using the GE 96x96 standard v1 program. For quality control, quantitative PCR (qPCR) amplification curves were first validated using gene expression Fluidigm BioMark real-time PCR analysis software on a Biomark (Fluidigm).

RNA-Seq. Briefly, peripheral MAIT cells were sorted in bulk (1,911- 64,011 total cells) using a FACS Aria SORP (BD Biosciences), pelleted, and overlaid with 250 μL of RNAlater (ThermoFisher) and frozen at -20°C. RNA-seq libraries were prepared using the SMART-Seq v4 Ultra Low Input RNA Kit (Clonetech) according to the manufacturer's instructions. Amplified material was purified using Agencourt AMPure XP beads (Beckman). cDNA quantity was assessed on a Qubit 3.0 (ThermoFisher) and fragment size was evaluated on a 2100 BioAnalyzer (Agilent). The PCR products were next indexed using the Nextera XT DNA Library Prep Kit (Illumina) according to the manufacturer's instructions. Briefly, products were tagmented using the Amplicon tagment mix containing Tn5 transposase, and indexed using Nextera index 1 (i7) and index 2 (i5) primers. The libraries were again cleaned-up with Agencourt AMPure XP beads, pooled, quantified, and sequenced across 75 base pairs (bp) using a single-end strategy with a 75-cycle high output flow

cell on a NextSeq 550 (Illumina). Nine biological replicates were sequenced per experiment, with 4 donor-matched time points corresponding to one pre-infection and three post HIV-infection time points at peak viral load, set point viral load, and early chronic infection. Median reads per sample was 22.9 million reads. GSEA analysis was generated using Broad Institute software (software.broadinstitute.org/gsea/index.jsp). Six of the donors with the highest sorted cell count across all four time points were subjected to MiXCR software for TCR analysis (https://mixcr.readthedocs.io/en/master/index.html). CDR3α sequence similarity analysis was performed with “MAIT Match” (http://www.cbs.dtu.dk/services/MAIT_Match), a tool where a score of 1 reflects a perfect match and a score of 0 a perfect mismatch with published MAIT cell CDR3α sequences. All CDR3α clones identified received a score of 1.

Supplementary references

1. K. G. Lal, E. Leeansyah, J. K. Sandberg, M. A. Eller, OMIP-046: Characterization of invariant T cell subset activation in humans. *Cytometry A*, (Mar 13, 2018).
2. J. Dias, J. K. Sandberg, E. Leeansyah, Extensive Phenotypic Analysis, Transcription Factor Profiling, and Effector Cytokine Production of Human MAIT Cells by Flow Cytometry. *Methods Mol Biol* **1514**, 241 (2017).
