# Supplementary material for: Dynamic MAIT cell response with progressively enhanced innateness during acute HIV-1 infection
Source: Nat Commun. 2020 Jan 14;11:272. doi: 10.1038/s41467-019-13975-9 (PMC6959336; doi:10.1038/s41467-019-13975-9)
Supplement: Supplementary file 3 — Reporting Summary [file 41467_2019_13975_MOESM3_ESM.pdf]

nature research

Corresponding author(s): Johan K. Sandberg

Last updated by author(s): Dec 3, 2019

Reporting Summary

Nature Research wishes to improve the reproducibility of the work that we publish. This form provides structure for consistency and transparency in reporting. For further information on Nature Research policies, see Authors & Referees and the Editorial Policy Checklist .

Statistics

For all statistical analyses, confirm that the following items are present in the figure legend, table legend, main text, or Methods section.

n/a	Confirmed	
☐	☒	The exact sample size (*n*) for each experimental group/condition, given as a discrete number and unit of measurement
☐	☒	A statement on whether measurements were taken from distinct samples or whether the same sample was measured repeatedly
☐	☒	The statistical test(s) used AND whether they are one- or two-sided<br>*Only common tests should be described solely by name; describe more complex techniques in the Methods section.*
☒	☐	A description of all covariates tested
☐	☒	A description of any assumptions or corrections, such as tests of normality and adjustment for multiple comparisons
☐	☒	A full description of the statistical parameters including central tendency (e.g. means) or other basic estimates (e.g. regression coefficient) AND variation (e.g. standard deviation) or associated estimates of uncertainty (e.g. confidence intervals)
☐	☒	For null hypothesis testing, the test statistic (e.g. *F*, *t*, *r*) with confidence intervals, effect sizes, degrees of freedom and *P* value noted<br>*Give P values as exact values whenever suitable.*
☒	☐	For Bayesian analysis, information on the choice of priors and Markov chain Monte Carlo settings
☒	☐	For hierarchical and complex designs, identification of the appropriate level for tests and full reporting of outcomes
☒	☐	Estimates of effect sizes (e.g. Cohen's *d*, Pearson's *r*), indicating how they were calculated

Our web collection on statistics for biologists contains articles on many of the points above.

Software and code

Policy information about availability of computer code

Data collection	Flow cytometry and transcriptional data was collected using publicly available software as described in the methods section of the manuscript.
Data analysis	Flow cytometry and transcriptional data was analyzed using publicly available software as described in the methods section of the manuscript.

For manuscripts utilizing custom algorithms or software that are central to the research but not yet described in published literature, software must be made available to editors/reviewers. We strongly encourage code deposition in a community repository (e.g. GitHub). See the Nature Research guidelines for submitting code & software for further information.

Data

Policy information about availability of data

All manuscripts must include a data availability statement. This statement should provide the following information, where applicable:

- Accession codes, unique identifiers, or web links for publicly available datasets
- A list of figures that have associated raw data
- A description of any restrictions on data availability

The authors declare that the data supporting the findings of this study are available within the article and its supplementary information files or are available upon reasonable requests to the authors. The RNA-Seq data have been deposited in NCBI's Gene Expression Omnibus and are accessible through GEO accession number GSE126752: [https://www.ncbi.nlm.nih.gov/geo/query/acc.cgi?acc=GSE126752]. The source data underlying medians in Figures 1b, c and g; 2a and b; 5d, 6d and 7a are provided as a source data files.

Field-specific reporting

Please select the one below that is the best fit for your research. If you are not sure, read the appropriate sections before making your selection.

- [x] Life sciences
- [ ] Behavioural & social sciences
- [ ] Ecological, evolutionary & environmental sciences

For a reference copy of the document with all sections, see nature.com/documents/nr-reporting-summary-flat.pdf

Life sciences study design

All studies must disclose on these points even when the disclosure is negative.

Sample size	The cases presented in this study are a selected set from a group of 29 RV217 participants, as well as samples from 7 acutely HIV-1 infected participants from the RV254/SEARCH 010 study sampled before initiation of cART, and 17 uninfected matched controls from the RV304/ SEARCH 013 study. Number of donors studied were not based on sample size calculations, but on availability of samples from the unique RV217 ECHO cohort study.
Data exclusions	No data was excluded from analysis.
Replication	The cases presented in this study are a selected set from a group of RV217 participants for which cryopreserved PBMC were available at pre-infection, and at least three post-infection time points corresponding to peak viral load (median 16 days since first positive test for HIV-1 RNA), set point viral load (median 43 days since first positive test for HIV-1 RNA), and early chronic infection (median 85 days since first positive test for HIV-1 RNA). Occasionally, long term follow up samples were used (out to 1,040 days since first positive test for HIV-1 RNA). Additional cross sectional studies were performed with samples from 7 acutely HIV-1 infected participants from the RV254/SEARCH 010 study sampled before initiation of cART, and 17 uninfected matched controls from the RV304/SEARCH 013 study (Supplementary Table 2).
Randomization	The subjects studied are among those that became infected during the longitudinal study of high risk individuals enrolled in the RV217 ECHO and RV254 Search010 studies. No additional randomization was undertaken.
Blinding	There was no blinding of samples during the course of these studies.

Reporting for specific materials, systems and methods

We require information from authors about some types of materials, experimental systems and methods used in many studies. Here, indicate whether each material, system or method listed is relevant to your study. If you are not sure if a list item applies to your research, read the appropriate section before selecting a response.

Materials & experimental systems

n/a	Involved in the study	
☐	☒	Antibodies
☒	☐	Eukaryotic cell lines
☒	☐	Palaeontology
☒	☐	Animals and other organisms
☐	☒	Human research participants
☒	☐	Clinical data

Methods

n/a	Involved in the study	
☒	☐	ChIP-seq
☐	☒	Flow cytometry
☒	☐	MRI-based neuroimaging

Antibodies

Antibodies used	See Supplementary Table 8 for all antibodies used.
Validation	All antibodies underwent an 8-point titration on PBMC from an HIV uninfected donor. Flow cytometry panel performance and functional assays were confirmed using a healthy donor before staining HIV infected donors from RV217 and RV254. Part of the optimization efforts are described in the published OMIP-046 in Cytometry part A by Lal et al. in 2018 (also referenced in the present manuscript).

Human research participants

Policy information about studies involving human research participants

Population characteristics	See Supplementary Tables 1 and 2
Recruitment	Briefly, the RV217 study enrolled high-risk, consenting adults at four clinical research sites: Walter Reed Project, Kericho, Kenya; Makerere University Walter Reed Project, Kampala, Uganda; Mbeya Medical Research Center, Mbeya, Tanzania; and Armed Forces Research Institute of Medical Sciences, Bangkok, Thailand. More information on RV217 cohort recruitment can be found at: Robb, M. L. et al. Prospective Study of Acute HIV-1 Infection in Adults in East Africa and Thailand. N Engl J Med 374, 2120-2130 (2016).<br>Information on RV254 cohort recruitment can be found at: Ananworanich, J. et al. Impact of multi-targeted antiretroviral treatment on gut T cell depletion and HIV reservoir seeding during acute HIV infection. PLoS One 7, e33948 (2012).
Ethics oversight	All subjects in studies RV217/WRAIR#1373, RV254/SEARCH 010/WRAIR#1494, and RV304/SEARCH 013/WRAIR#1751 were adults and provided written informed consent. For subjects that were unable to read, the consent document was read to them with an impartial witness present; the volunteer, the witness and the study staff obtaining consent signed the affidavit with a signature or mark. Studies were reviewed and approved by the human subject ethics and safety committees in each country, as well as by the Walter Reed Army Institute of Research (WRAIR) (Silver Spring, MD, USA), in compliance with relevant federal guidelines and institutional policies. RV304: The Institutional Review Board of the Faculty of Medicine, Chulalongkorn University; and the WRAIR Institutional Review Board. RV217: Institutional Review Board Royal Thai Army Medical Department; Kenya Medical Research Institute (KEMRI) Scientific and Ethics Review Unit (SERU); Uganda National HIV/AIDS Research Committee (NARC); Mbeya Medical Research and Ethics Committee (MMREC) and the National Health Research Ethics Committee (NatHREC); and the WRAIR Institutional Review Board. RV254: The Institutional Review Board of the Faculty of Medicine, Chulalongkorn University; and the WRAIR Institutional Review Board.

Note that full information on the approval of the study protocol must also be provided in the manuscript.

Flow Cytometry

Plots

Confirm that:

- [x] The axis labels state the marker and fluorochrome used (e.g. CD4-FITC).
- [x] The axis scales are clearly visible. Include numbers along axes only for bottom left plot of group (a 'group' is an analysis of identical markers).
- [x] All plots are contour plots with outliers or pseudocolor plots.
- [x] A numerical value for number of cells or percentage (with statistics) is provided.

Methodology

Sample preparation	Four polychromatic flow cytometry-based panels were used to measure MAIT cell function, phenotype, and for cell sorting for transcriptomics. Briefly, thawed samples were washed, stained with LIVE/DEAD Fixable Aqua Dead Cell dye (ThermoFisher), blocked for Fc receptors using Normal mouse serum (ThermoFisher), and surface stained with antibody cocktail. Samples were surface stained at room temperature for 30 minutes, and some were intracellularly stained at room temperature for 30 minutes. Some samples were fixed in 2% paraformaldehyde or BD FIX/PERM Buffer (BD Biosciences). Other samples used for sorting for downstream transcriptomics and were resuspended in sorting buffer (PBS containing 1% BSA) and sorted for bulk MAIT cells for either RNA-Seq or targeted transcriptomics. Peripheral MAIT cells were purified by sorting (1,911 - 64,011 total cells) using a FACS Aria SORP (BD Biosciences), pelleted, and overlaid with 250 µl of RNAlater (ThermoFisher) and frozen at -20°C. RNA was extracted using the RNeasy Mini Kit (Qiagen), and RNA quality and concentration were assessed with the Agilent 2100 Bioanalyzer Pico Chip. RNA-Seq libraries were prepared using the SMART-Seq v4 Ultra Low Input RNA Kit (Clontech) according to the manufacturer's instructions. Amplified material was purified using Agencourt AMPure XP beads (Beckman). cDNA quantity was assessed on a Qubit 3.0 (ThermoFisher) and fragment size was evaluated on a 2100 BioAnalyzer (Agilent). The PCR products were next indexed using the Nextera XT DNA Library Prep Kit (Illumina) according to the manufacturer's instructions. Briefly, products were tagmented using the Amplicon tagment mix containing Tn5 transposase, and indexed using Nextera index 1 (i7) and index 2 (i5) primers. The libraries were again cleaned-up with Agencourt AMPure XP beads, quantified, pooled, and sequenced across 75 base pairs (bp) using a single-end strategy with a 75-cycle high output flow cell on a NextSeq 500 (Illumina). Other samples were stimulated for either 24 hours with partially fixed E. coli D21 in the presence of anti-CD28, for 24 hours with IL-12 and IL-18 as previously described, or for 6 hours with PMA/ionomycin as per the manufacturer's recommendation (eBioscience™ Cell Stimulation Cocktail (500X), ThermoFisher). All stimulation methods included BFA and monensin for the last 6 hours of stimulation.<br>Sampling of gut-associated lymphoid tissue was performed by sigmoidoscopy, and mucosal mononuclear cells (MMCs) were isolated as described. Briefly, 20–25 pieces of gut-associated lymphoid tissue were collected from the sigmoid colon by sigmoidoscopy using Radial Jaw 3 biopsy forceps (Boston Scientific, Natick, MA, USA). The biopsy pieces were placed in complete RPMI 1640 RPMI media containing 10% human AB serum (HAB; Gemini Bio-Product, West Sacramento, CA, USA), 1% HEPES, 1% L-Glutamine, 0.1% Gentamicin (Invitrogen, Carlsbad, CA, USA), 1% Penicillin/Streptomycin and 2.5 µg/ml Amphotericin B (Invitrogen, Carlsbad, CA, USA). Samples were then digested using 0.5 mg/ml Collagenase II (Sigma, St. Louis, MO, USA). Isolated MMCs from one donor were pooled, washed twice and then counted using Trypan Blue exclusion. MMCs were directly used for phenotypical characterization by flow cytometry.<br>Luminex® based detection assays were used to measure plasma levels of C-reactive protein (CRP) and IL-6 (EMD Millipore, Billerica MA) per manufacturer's instructions. Briefly, samples were mixed with a cocktail of MagPlex® magnetic microspheres,

bound to capture antibody specific to proteins of interest. Following incubation with sample overnight at 4°C, excess sample was washed off using a magnetic plate washer (BioTek Instruments, Winooski VT) and biotinylated detection antibody cocktail was added for 1 hour at room temperature. Streptavidin-phycoerythrin was added for 30 minutes before a final wash and resuspension in sheath fluid. Data was collected on a FlexMap 3D® system. Levels of sCD14 and intestinal fatty acid binding protein (IFABP) were measured by standard chemiluminescent detection ELISA (R&D Systems, Minneapolis MN) per manufacturer's instructions and read on a VersaMax® reader (Molecular Devices, Sunnyvale CA).

Instrument

BD LSRII SORP flow cytometer (BD Biosciences), BD ARIA SORP cell sorter (BD Biosciences), Biomark HD (Fluidigm), NextSeq 500 (Illumina)

Software

FlowJo v.9.9.4 (TreeStar), Prism version 6.0 for Mac OS X (GraphPad, La Jolla CA), Real-Time PCR Analysis software (BioMark). The Unix based program, STAR (PMID:23104886) (Spliced Transcripts Alignment to a Reference) v.2.6.1 with human genome hg38, was used for alignment. Transcription mapping was performed using RSEM (PMID: 21816040) (RNA-seq by expectation Maximization) v.1.3.1. The featureCounts (PMID: 24227677) program was used for counting mapped reads. RUVSeq (PMID: 25150836) v1.12 was used to remove unwanted variation, and differentially expressed gene list was generated by edgeR (PMID: 19910308) v3.20. R package. GSEA (PMID:16199517)(Gene Set Enrichment Analysis) method was used for finding statistically significant pathways with 5917 gene sets of GO (gene oncology) in MSigDB (Molecular Signatures Database) issued by Broad Institute. TCR data was extracted from the RNA-Seq dataset from six donors with sufficiently high cell counts across all four analyzed time points, using the MiXCR software (https://mixcr.readthedocs.io/en/master/index.html).

Cell population abundance

MAIT cells identified as double positive for CD161++Va7.2+ had a frequency ranging between 0.17%-11.9% of total T cells.

Gating strategy

See Supplementary Figure 1. Generally, MAIT cells were identified as events with consistent fluorescence (TCR Va7.2+ vs. time), single cells (FSC-H vs. FSC-A), uniform scatter profile (SSC-A vs. FSC-A), living (SSC-A vs. Aqua LIVE/DEAD), CD3+ non-macrophages and non-B cells (CD3 vs. CD14/CD19 dump channel), and co-expressing CD161++Va7.2+. MAIT cells were confirmed to be MR1 restricted by staining in some donors for co-expression of Va7.2+ and MR1 5-OP-RU tetramer amongst CD3+ T cells, with >96% overlap between MAIT cells identified as CD161++Va7.2+ amongst CD3+ T cells.

☒ Tick this box to confirm that a figure exemplifying the gating strategy is provided in the Supplementary Information.
